# Structure and Raman Spectra of Exotic Carbon Microcrystals from Meteoritic Dust of Chelyabinsk Superbolide

**DOI:** 10.3390/nano13010073

**Published:** 2022-12-23

**Authors:** Galina Savosteenko, Sergey Taskaev, Pavel Avramov

**Affiliations:** 1 Physical Department, Chelyabinsk State University, Chelyabinsk 454001, Russia; 2 Energy Efficiency Research and Education Center, University of Science and Technology “MISIS”, Moscow 119049, Russia; 3 College of Natural Sciences, Department of Chemistry, Kyungpook National University, Daegu 41566, Republic of Korea

**Keywords:** Chelyabinsk superbolide, meteoritic dust, multiply twinned closed-shell graphite microcrystals

## Abstract

The Chelyabinsk superbolide, the largest in XXI century, which exploded on 15 February 2013 over snowy fields of Southern Urals was a historic event not just only because of its massive scale and explosive power. High-temperature, high-pressure conditions in the front shock wave caused intense ablation of the asteroid material and formation of huge amount of meteoritic dust dispersed in the atmosphere during the flyby. Massive snowfalls just few days before and after the event conserved precipitated meteoritic dust in thin layer of snow which was collected and studied later. The most intriguing and challenging material discovered in the dust was closed-shell 10–70 µm exotic polygonal graphitic carbon microcrystals of undisclosed nature. Using optical and electron microscopy and Raman spectroscopy the atomic structure of closed-shell microcrystals was thoroughly studied and their graphitic nature was revealed. It was found that some of the particles formed by multilayer graphitic polygonal shells have extensive hollows inside. Comparative microscopic and spectroscopic analysis of meteorite exotic carbon microcrystals with different graphite species, carbon onions, and diamond revealed two distinctively different closed-shell carbon particles. The first type of the particles can be attributed to carbon onions with characteristic graphite nanocrystalline basic structural units (BSU) of 49 nm lateral size and less and, probably, BSU heteroatomic termination necklace with oxygen content comparable to 1.1% and more. It was shown that the second type of unique graphitic carbon particles of a convex shape and perfect hexagonal symmetry with lateral dimensions of 14 µm correspond to multiply twinned closed-shell graphite microcrystals with polyhexacyclooctadecane (–C_18_–)_n_ core wrapped by multiple layers of carbon honeycombs with low (<1%) content of oxygen termination necklace.

## 1. Introduction

Chelyabinsk (Russia) superbolide [1] was a unique phenomenon of enormous scale. The parent asteroid which entered the Earth atmosphere on 15 February 2013, had an initial diameter about 18 m, mass around 12,000 metric tons and caused several atmospheric blasts with total energy release up to 500 kt of TNT, which is approximately 15 times larger than the total energy release of the atomic bombs exploded in Hiroshima and Nagasaki back in 1945. It was the biggest meteoroid in the 21st century and just the second one after the Tunguska event, 1908, Russia.

The Chelyabinsk superbolid was a historical event due to different reasons. Every year several thousand tons of space debris fall to Earth in the form of micrometeorites. Normally, they are fully or partially remelted, evaporated and condensed back in the atmosphere to form dust particles with diameters less than 50 μm [2,3,4]. Despite massive meteoritic dust annual precipitate, collection of such materials is a very challenging job because of extremely low specific concentrations in the air and on the Earth surface. In fact, the dust is just a natural material mostly of silicate nature, so it is possible to separate it from the environment only in rare locations like swamps, deserts, beach sand, deep-sea sediments and sedimentary rocks, and different types of glacier ice [5,6,7,8,9,10,11,12,13,14,15,16].

Almost all 12,000 t of parent Chelyabinsk asteroid material—ordinary chondrite of the LL5 petrological class [1]—was pulverized in the atmosphere during superbolide 280 km flyby trajectory with the dust plume formed at altitudes from 80 to 27 km above the Earth surface [17]. The weather conditions in which the meteoritic dust fell after the atmospheric flyby was exceptional to conserve the material to be collected later [18]. Just 8 days before the event there was a snowfall in which meteoritic dust was collected. This snow layer created a distinct borderline allowing determination of the layer’s beginning. Another snowfall 13 days after the meteorite’s fall conserved the meteorite dust that had fallen out by that time.

Among meteorite materials collected at the end of winter–beginning of spring 2013 from snowy Southern Urals steppes, the most intriguing and challenging were exotic carbon microcrystals of undisclosed nature formed by reduction and precipitation of elementary carbon from atmospheric CO_2_ on chondrite particles [18]. It was found that under extreme high-temperature, high-pressure conditions of meteorite front shock wave either elongated or non-elongated 10 to 100 µm exotic multifaceted polygonal closed-shell carbon microcrystals were formed and most of them fused in irregular complex conglomerates.

Based on microscopic, structural and spectroscopic data as well as theoretical quantum-chemical and molecular-dynamics simulations multiply twinned closed-shell graphite microcrystals formed during structural decomposition of Goldberg-type nano- and microdiamonds [19,20] were hypothesized [18] as possible structural models of multifaceted polygonal closed-shell exotic carbon microcrystals (ECM) of mesoscopic dimensions. Current study provides further detailed and important insights of structure and properties of the microcrystals based on new microscopic images of the dust carbon phase, detailed description of sample preparation procedure, detailed interpretation of Raman spectra of the key samples of multiply twinned closed-shell graphite microcrystals, and comparative analysis of the Raman spectra of Chelyabinsk particles with previously published spectra of different graphites, diamond and carbon onions.

The main goal of this study is a detailed microscopic and Raman spectroscopy study of unique exotic multifaceted carbon microcrystals of complex nature discovered in Chelyabinsk meteoritic dust precipitated from the atmosphere and conserved in deep Ural snow back in February of 2013. For the first time, key details of sample preparation of meteoritic dust which contains ECMs are presented in the study. Using optical and electron microscopy and Raman spectroscopy it was discovered that exotic multifaceted microcrystals are *sp*^2^ closed-shell graphitic particles with distinctive hollows inside. It was shown that the multilayered graphitic walls of some microcrystals are formed by fused triangular fragments which form pentagonal and hexagonal facets of finite dimensions. For the first time original extended collection of Raman spectra of Chelyabinsk meteorite carbon particles are presented in the study. Based on comparative analysis of the images and Raman spectra, two very distinctive types of multiply twinned ECMs were identified. Comparative analysis of Raman spectra of the first type of ECM with different graphite species, diamond, and carbon onions attributed ECM to carbon onions with characteristic graphite nanocrystalline basic structural units (BSU) of 49 nm lateral size and less and, probably, BSU heteroatomic termination necklace with oxygen content comparable to 1.1% or more. A comparative analysis of microscopic images and Raman spectra of unique graphitic carbon particle of perfect hexagonal symmetry with lateral dimensions up to 14 µm directly indicates an existence of previously hypothesized perfect multiply twinned closed-shell graphite microcrystals with polyhexacyclooctadecane (–C_18_–)_n_ core [18] and low (<1%) content of BSU oxygen termination necklace.

## 2. Preparation of Carbon Samples

**Figure 1:**
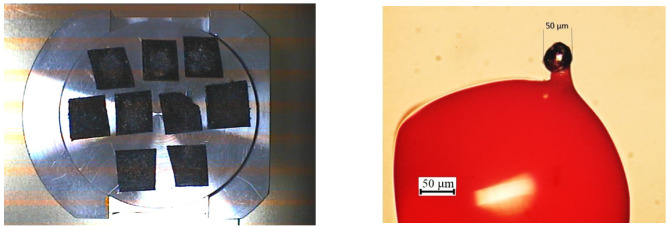
(**left**) Samples of meteoritic dust of Chelyabinsk meteorite in vacuum chamber of electron microscope. (**right**) Optical image of recovered carbon particle presented in Figure 2a bottom-right glued on epoxy resin.

The collection of meteorite dust turned out to be possible due to favorable meteorological conditions, namely extensive anticyclone with cloudy weather without any fallout within a few days after the meteorite flyby. As a result, the snow hardened, and an icy crust formed on its surface. In total, 53 samples were collected in trajectory region. The snow was melted under in a warm room, and the resulting water was filtered through a funnel with filter paper placed in it and meteorite dust samples were collected (Figure 1 (left)). The mass of the finely dispersed substance obtained by filtration is in the range from 0.0096 g to 0.24 g per sample.

The meteorite dust samples were glued to the adhesive tape not to allow the particles to scatter during pumping out air in the electron microscope. Despite the small particle diameters ~50 μm, the ECM samples from the adhesive tape which displayed challenging crystal surface patterns were glued on cactus needles to make high-quality microscopic images (Figure 1 (right)). Then, the cactus needles were embedded in a special compound to create a rigid connection with the manipulator.

## 3. Microscopy Images of Carbon Samples

**Figure 2:**
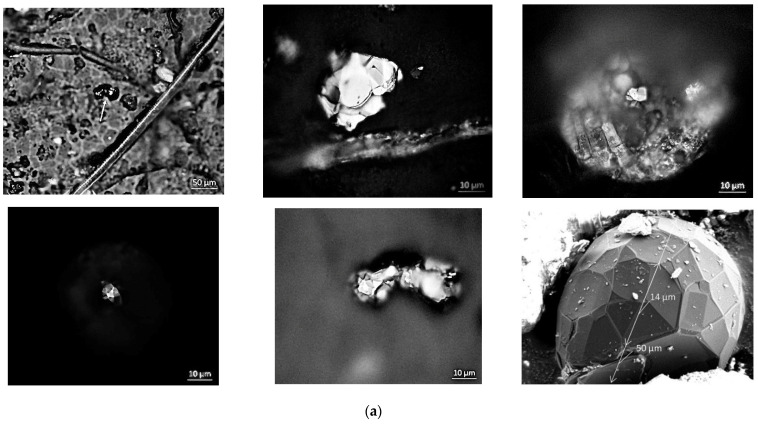

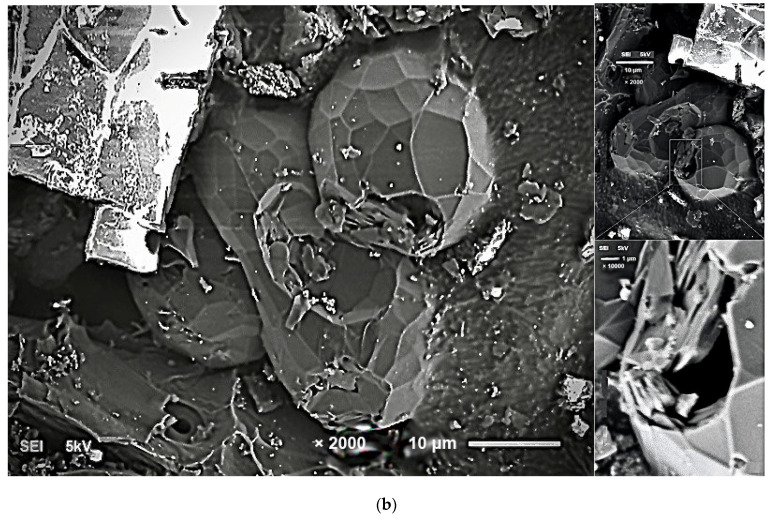
(**a**) Optical images of carbon particles in the dust of Chelyabinsk superbolide. Complex multifaceted polygonal nature of the particles is clearly seen in all images. In the bottom-right image the effective dimensions of 50 µm multifaceted exotic microcrystal are indicated by arrows. (**b**) Scanning electron microscopy images of a conglomerate of complex polygonal exotic carbon microcrystals (ECM), including elongated particle (top, 2000 magnification) and zoomed bottom (10,000 magnification) rectangular area in top image. In the bottom image the multilayer graphitic nature of the particle and an extensive hollow inside the particle are clearly seen.

**Figure 3:**
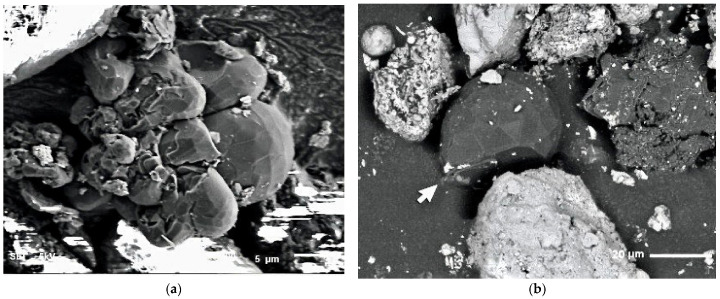
(**a**) Scanning electron microscopy image of a conglomerate of complex multifaceted polygonal ECM with 2000 magnification. (**b**) Scanning electron microscopy image of a complex multifaceted polygonal ECM with indented wall (2000 magnification).

**Figure 4:**
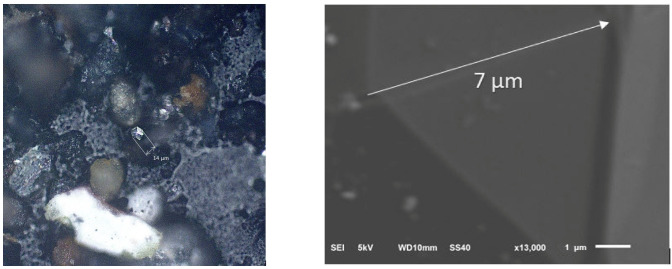
On the (**left**): Optical image of hexagonal ECM particle of 14 μm maximum dimension. Right: Electron microscopy image of one 7 μm edge of hexagonal particle presented on the (**right**).

Optical and electron microscopy images of several samples of meteorite exotic carbon microcrystals discovered in Chelyabinsk meteorite dust [18] are presented in Figure 2, Figure 3 and Figure 4. Both optical (Figure 2a) and electron (Figure 2b) images clearly demonstrate ECM complex polygonal nature. In Figure 2a the optical images of the particles clearly demonstrate complex conglomerates formed by multifaceted polygonal ECMs with effective dimensions up to 50 µm. In Figure 2b, top and bottom, scanning electron microscope images of a conglomerate of exotic polygonal elongated and non-elongated 20–50 µm ECMs are presented with 2000 and 10,000 magnification, respectively. Triagonal, tetragonal, pentagonal, hexagonal, and, probably, even higher-gonal facets are clearly seen in the images of the particles. Just closer to the bottom of the center of Figure 2b image, a rectangular area reveals a crashed region of polygonal non-elongated ECM.

In Figure 2b bottom zoomed rectangular region is presented with 10,000 magnification. A layered structure of the wall of the particle with the thickness of approximately 1 µm is clearly seen with extended hollow inside. Elongated polygonal ECM in Figure 2b top also demonstrate clear indentation just in its center, which could be considered as a witness of the hollow inside the particle.

Scanning electron microscopy images (Figure 3a,b) clearly reveal multifaceted polygonal ECMs of complex shapes and dimensions in the range from 5 to 40 µm with crashed walls and indentations of different shapes and depth. Following the images, the particles which consist of convex graphitic polygons, mostly of hexagonal shape, may contain complex in nature twin structures formed by triangular facets.

The most intriguing 14 µm particle of hexagonal symmetry with six top facets is clearly seen in the center of optical image of Figure 4 left. On the right of Figure 4 enlarged 13,000 zoomed electron microscopy image of one edge of 7 µm length of the hexagonal particle from Figure 4 left is presented. Perfect polygonal character of the particle is clearly seen on both images. In fact, the symmetry and linear dimensions of this ECM is very much alike the central hexagonal fragment of the complex multifaceteded ECM from bottom-right of Figure 2a with linear dimension of hexagonal fragment also equal to 14 µm.

## 4. Raman Spectra of Carbon Species

**Figure 5:**
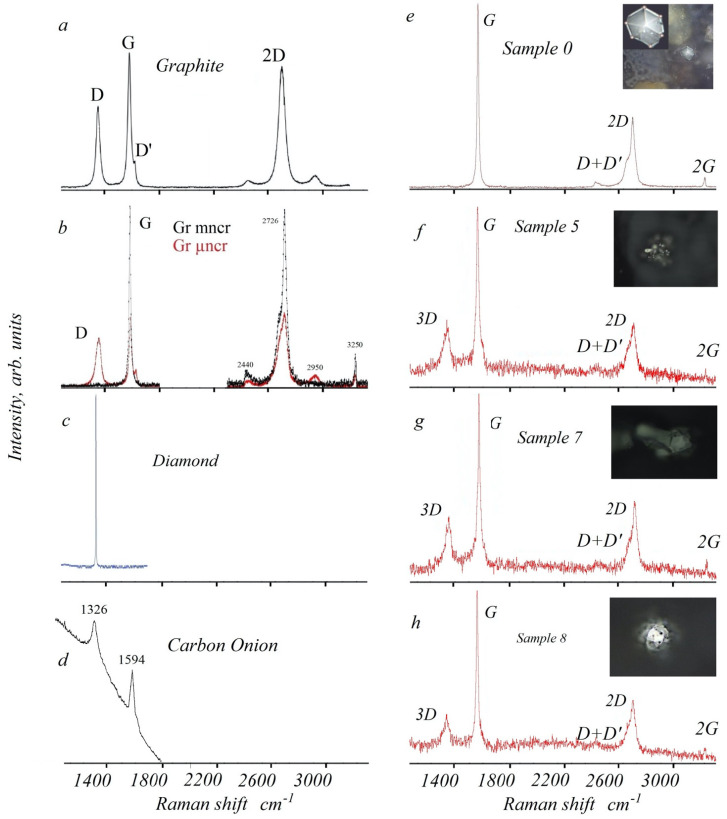
(**a**): Typical Raman spectrum of graphite; (**b**): Raman spectra of Mono- (*mncr*) (in black) and micronanocrystalline (*μncr*) (in red) graphites [21,27,28,29]; (**c**): Typical diamond Raman spectrum; (**d**): Raman spectrum of carbon onion [26] (Adapted with permission from Ref. [26], 2000, Elsevier). (**e**): Chelyabinsk superbolid ECM Sample 0 Raman spectrum. In the insert, a hexagonal particle from the center of Figure 1a bottom-right is presented; (**f**): Sample 5 Raman spectrum; (**g**): Sample 7 Raman spectrum; (**h**): Sample 8 Raman spectrum. The optical images of ECM particles (2000 magnification) are presented in the inserts for Chelyabinsk superbolid ECM samples.

Raman spectroscopy (RS) is a powerful tool to study structural properties of different carbon allotropes. One can easily found detailed reviews devoted to RS of different carbon species (see, for example, [21,22,23,24]) with detailed descriptions of spectral formation mechanisms and the key spectroscopic features of different carbon species. One of the key publications in the field of Raman spectroscopy of carbon species was a study of Tuinstra et. al. [25] devoted to graphite RS spectra in which a wide peak of polycrystalline graphite with the energy of 1355 cm^−1^ was attributed to the *sp*^2^ BSU of 49 nm letteral size and less, rather than to strong and sharp peak of *sp*^3^ carbon atoms. Raman spectra were also used to characterize carbon fullerenes and nanotubes [22], giant fullerenes and carbon onions [26], shungites and anthraxolites [23] etc. Raman spectra of *sp*^2^ carbon species (see, for example [21]) is divided into two regions, namely One-phonon region with characteristic frequencies of 1100–1700 cm^−1^ and Two-phonon region with characteristic frequencies of 2300–3500 cm^−1^, each of which display several spectral features responsible for different structural characteristics of the species. The main goal of this study is to analyze the Raman spectra of Exotic Carbon Microcrystals discovered in meteoritic dust of Chelyabinsk meteorite using Thermo Fisher Scientific Raman spectrometer at Chelyabinsk State University with green laser 532 nm excitation on 1 mW power with 10 s exposition time to avoid significant heating of the object. The studies were carried out at room temperature on several objects, the optical images of which are shown in the insets of Figure 5. The laser was focused on the center of the face from which the signal was taken.

The Raman spectra of Chelyabinsk meteorite ECMs are presented in Figure 5e–h, with exact positions and widths of the peaks in Table 1. Two main peaks located in the regions approximately 1582 and 2750 cm^−1^ can be distinguished in the spectra of all ECMs. The peak at 2750 cm^−1^ is the second order *D* peak and is called the 2*D* mode (Disorder, defects) and is presented even in high-quality graphite samples (see below). The red shoulder of 2*D* peak maybe attributed to some structural defects as well. The second peak (1582 cm^−1^) is called *G* mode (Graphitization) and shows the degree of graphitization. The presence of 3*D* peak at a frequency of ~1330 cm^−1^ in all but one (Sample 0) RS ECMs can be caused by multiple structural defects, facets, etc. and it will be discussed in details below. It is necessary to note that it is very different from Raman intensity caused by *sp*^3^ hybridized carbon atoms. 2*G* and *D* + *D*’ peaks at 2950 and 2950 cm^−1^ are attributed to high-quality graphite as well could be determined by anharmonicity caused by electric factor [21].

## 5. Discussion

Let’s consider first RS one-phonon spectral region (1100–1700 cm^−1^) of graphite-related materials (Figure 5). It is well known [25,27,28] that Raman spectra of the best monocrystalline graphites (for example, Madagascar, Ticonderoga, Ceylon, and Botogol’sk) are characterized by a single *G*-band spectrum in one-phonon spectral region. The *G* peak [25] at 1575 cm^−1^ was attributed to Raman-active E_2g_ mode of perfect graphite monocrystal. Comparison of the *D* mode wide peak with half width ~40 cm^−1^ at 1355 cm^−1^ with Raman spectrum of diamond (very narrow line at 1332 cm^−1^) somehow excludes a possibility of a significant contribution of *sp*^3^ coordinated atoms in formation of Raman spectra in one-phonon region of any *sp*^2^ species. Following [25], wide *D* mode (3*D* mode for Chelyabinsk ECMs, Table 1) of polycrystalline graphite was interpreted as a manifestation of a change in the selection rules for Raman activity of certain phonons which are inactive in the infinite lattice. Due to the size effect, for small crystallites the Raman activity in this region can be observed. So, the *D* mode is a manifestation of A_lg_ mode of small crystallites, or boundaries of larger crystallites with the intensity inversely proportional to the effective crystallite dimensions. The activation of A_1g_ mode in Raman spectra is caused by finite crystal size. In general, the intensity of *D* band allows one to measure the effective crystallite lateral size *L_a_* in a thin surface layer of any carbon sample (see typical RS spectrum of graphite, Figure 5a). The graphite spectrum is also characterized by visible *D*’ peak with the energy 1600–1620 cm^−1^, which reflects *sp*^2^ surface defects of graphitic microcrystals.

The one-phonon Raman spectra of mono- (mncr) and micronanocrystalline (µncr) graphites [29] are presented in Figure 5b. Like Figure 5a graphite spectrum, the µncr-graphite RS displays both wide *D* and *D*’ peaks which reflect polycrystalline and defect nature of BSUs which form the µncr sample. It was found [21], that the effective crystallite dimension *L_a_* for µncr-graphite is equal or less 49 nm with necklace composition of oxygen up to 1.1% and higher. In contrast to it, the mncr graphite Raman spectrum has no either *D* or *D*’ peaks, which directly indicate the *L_a_* longer 105 nm without any *sp*^2^ surface defects and concentration of necklace oxygen of 1.0% and less.

Diamond Raman spectrum (Figure 5c) is presented for the sake of comparison.

The one-phonon Raman spectrum of giant fullerenes and carbon onions [26] (Adapted with permission from Ref. [26], 2000, Elsevier) is presented in Figure 5d. As for the spectra of polycrystalline graphites (see above), the spectrum is characterized by visible peaks in *D* and *D*’ energy regions which directly indicates small effective dimensions of structural fragments, *sp*^2^ surface defect states which could be caused by carbon pentagon and heptagon rings and, probably a presence of visible amount of terminated oxygen groups.

Let’s analyze one-phonon spectra of Chelyabinsk superbolid ECMs presented in Figure 5e–h and Table 1. In Figure 5e the Sample 0 RS is presented with enlarged optical image of hexagonal particle from Figure 4 in the insert. In one-phonon region Sample 0 RS displays only very sharp *G* peak (1582 cm^−1^) without any traces of either 3*D* peak, or blue shoulder of *G* peak (Table 1) which correspond to *D* and *D*’ peaks for graphites (Figure 5a–d). It directly indicates the existence of extended BSU lateral size *L_a_* of at least 7 µm without any traces of surface *sp*^2^ defects [21]. Raman spectra of Sample 0 ECM in one-phonon region satisfies structural features of proposed earlier graphite multiply twinned microcrystals of perfect hexagonal symmetry constituted by inserted into each other *sp*^2^ carbon honeycombs with central polyhexacyclooctadecane (–C_18_–)_n_ core [18,19,20].

RS spectra in one-phonon region of Samples 5, 7, 8 of Chelyabinsk ECMs (Figure 5f–h, Table 1) demonstrate extended peak in 3*D* region (1365 cm^−1^) and a visible blue shoulder of *G* peak (1582–1590 cm^−1^). These spectral features reveal multiple types of lattice defects (see above) and correspond to the lateral BSU size *L_a_* smaller 49 nm coupled with oxygen neckless concentration greater 1.1% and a presence of surface *sp*^2^ defects. Taking into account their closed-shell nature the spectra mostly resemble Raman spectrum of giant fullerenes and carbon onions presented at the bottom of Figure 5a–d.

The two-phonon Raman spectra (2500–3300 cm^−1^) of *sp*^2^ carbon materials [21,30] have no band overtones of one-phonon spectra and mostly reflect the anharmonicity of both vibrational and electronic states with predominant role of electric factor. One can speculate [31] that the highly delocalized character of *sp*^2^ π-electron system contributes to such pronounced anharmonicity with the shape [21] determined by the regularity and lateral dimensions of BSU. In contrast to highly-ordered mono-graphite specie (Figure 5b) [29], the RS of amorphous *sp*^2^ carbon materials in two-phonon spectral region demonstrate diffuse shapes with very wide peaks which intensity and width is mostly determined by the lateral dimensions of the fragments and structural irregularities.

The ECM Raman spectra in two-phonon spectral region of Chelyabinsk superbolid (Samples 0, 5, 7, 8, Figure 5e–h, combined with corresponded optical images from Figure 2, Figure 3 and Figure 4 as inserts) demonstrate two distinctly different shapes. The first one, Sample 0 Raman spectrum (Figure 5e, Table 1), demonstrate 3 spectral features (2446, 2719, and 3250 cm^−1^) which perfectly correspond to the 2440, 2726 and 3250 cm^−1^ peaks of mono-graphite [29] with extended BSU lateral dimensions which might correspond to twinned graphite closed-shell microcrystal with polyhexacyclooctadecane (–C_18_–)_n_ core [18,19,20].

The RS two-phonon region of Samples 5, 7, 8 (Figure 5f–h, Table 1) demonstrate typical graphite spectral features (2439–2450, 2722–2738, 3250 cm^−1^) with relatively small structural domains at 2440, 2726 and 2950 cm^−1^. It is necessary to note that the intensity ratio of *G*/2*D* peaks for Samples 5, 7, 8 is visibly smaller than the ratio for Sample 0 (Figure 5e) and perfectly correspond to relative *G*/2*D* peaks intensity ratio for µncr-graphites [29] with BSU size smaller 46 nm and high concentration of defects. Based on joint analysis of microscopic images and Raman spectra of Samples 5, 7, 8 one can conclude that the closed-shell ECM correspond to carbon onions [26,32].

## 6. Conclusions

Concerted comparative analysis of microscopic images and Raman spectra of Chelyabinsk meteorite exotic carbon microcrystals and different *sp*^2^ carbon materials like graphites and carbon onions revealed two distinctively different types of ECM particles discovered in meteorite dust. The first type, closed-shell graphitic carbon particles structurally and spectroscopically correspond to typical carbon onions. The second type of exotic carbon microcrystals [18], perfect closed-shell multiply twinned graphite microcrystals of hexagonal symmetry with structural domains > 46 µm is characterized by polyhexacyclooctadecane (–C_18_–)_n_ core which served as formation embryo during the high-temperature, high-pressure synthesis on chondrite particles from atmospheric CO_2_. Following structural model presented earlier [18], the ECM structural polyhexacyclooctadecane core is wrapped by multiple layers of carbon honeycombs which form perfect closed-shell multiply twinned graphite microcrystal with low (<1%) content of oxygen termination necklace.

## Figures and Tables

**Table 1:** Spectral line energy positions (and width) (cm^−1^) in the Raman spectra of Chelyabinsk meteorite ECMs.

Sample No	0	5	7	8
Peak Name				
3*D*	---	1365 cm^−1^ (100 cm^−1^)	1365 cm^−1^ (100 cm^−1^)	1364 cm^−1^ (100 cm^−1^)
*G*	1582 cm^−1^ (120 cm^−1^)	1590 cm^−1^ (110 cm^−1^)	1585 cm^−1^ (110 cm^−1^)	1582 cm^−1^ (100 cm^−1^)
*D* + *D*’	2446 cm^−1^	2450 cm^−1^	2439 cm^−1^	2450 cm^−1^
2*D*	2719 cm^−1^ (120 cm^−1^)	2738 cm^−1^ (120 cm^−1^)	2722 cm^−1^ (150 cm^−1^)	2729 cm^−1^ (150 cm^−1^)
2*G*	3250 cm^−1^	3250 cm^−1^	3250 cm^−1^	3250 cm^−1^

## Data Availability

The data that support the findings of this study are available upon reasonable request from the authors.
